# Why Do Sleeping Nematodes Adopt a Hockey-Stick-Like Posture?

**DOI:** 10.1371/journal.pone.0101162

**Published:** 2014-07-15

**Authors:** Nora Tramm, Naomi Oppenheimer, Stanislav Nagy, Efi Efrati, David Biron

**Affiliations:** 1 Department of Physics and the James Franck Institute, The University of Chicago, Chicago, Illinois, United States of America; 2 The Institute for Biophysical Dynamics, The University of Chicago, Chicago, Illinois, United States of America; Children Hospital of Philadelphia, United States of America

## Abstract

A characteristic posture is considered one of the behavioral hallmarks of sleep, and typically includes functional features such as support for the limbs and shielding of sensory organs. The nematode *C. elegans* exhibits a sleep-like state during a stage termed lethargus, which precedes ecdysis at the transition between larval stages. A hockey-stick-like posture is commonly observed during lethargus. What might its function be? It was previously noted that during lethargus, *C. elegans* nematodes abruptly rotate about their longitudinal axis. Plausibly, these “flips” facilitate ecdysis by assisting the disassociation of the old cuticle from the new one. We found that body-posture during lethargus was established using a stereotypical motor program and that body bends during lethargus quiescence were actively maintained. Moreover, flips occurred almost exclusively when the animals exhibited a single body bend, preferentially in the anterior or mid section of the body. We describe a simple biomechanical model that imposes the observed lengths of the longitudinally directed body-wall muscles on an otherwise passive elastic rod. We show that this minimal model is sufficient for generating a rotation about the anterior-posterior body axis. Our analysis suggests that posture during lethargus quiescence may serve a developmental role in facilitating flips and that the control of body wall muscles in anterior and posterior body regions are distinct.

## Introduction

The identification of sleep by researchers most commonly employs electrophysiological criteria [1]–[3]. These criteria were originally developed in mammalian species, relying on the observed close interdependence between electrophysiological brain activity and behavioral indicators of sleep, but they were not found to be generally applicable across phyla [4]. Sleep can more generally be defined using its behavioral indicators, which include: (1) a homeostatic drive, (2) behavioral quiescence, (3) an elevated arousal threshold, (4) reversibility, and (5) a species-specific stereotypical posture [4]–[10]. Sleep-postures have been described for numerous vertebrate and invertebrate species, and in many cases have been shown to correlate with elevated arousal thresholds [4]. Typically, they include functionally beneficial features such as support of a substrate for the limbs and the head, a hunched (protective) body posture, and shielding of sensory organs. A distinctive posture is thus considered a behavioral hallmark sleep.

**Figure 1:**
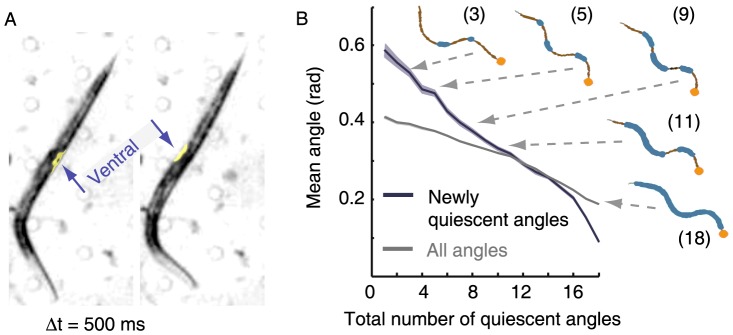
*C. elegans* enter the quiescent posture with stereotypical dynamics. (A) A hockey-stick-like posture containing a single body bend, commonly observed during lethargus quiescence bouts (see also Fig. 3) before (left) and after (right) a flip. The image on the right was captured 500 ms after the image on the left, by which time the flip had been completed. The arrows point to the ventral side of the animal in each of the two figures. (B) The average angle at positions along the body that were becoming quiescent as the animals were entering bouts of quiescence. Eighteen equidistant positions along the body were individually assayed for quiescence at every time-point during L4leth, and initiations of whole-animal bouts of quiescence were identified. The angles between adjacent segments of the midline at positions that newly became quiescent were compared to the mean of all 18 angles at the same time-point. Animals initially froze high-curvature body-positions and subsequently lower-curvature positions also became quiescent, indicating a stereotypical dynamics of entering a quiescence bout. In addition, five snapshots of midlines are shown, taken from a single event of entering a bout of quiescence. The orange circles depict the head of the animal, the thick blue lines depict the quiescent angles along each midline, the numbers in parenthesis indicate the total number of quiescent angles in each midline, and the arrows point to the corresponding position on the plot. The plot depicts data from N = 37 animals, mean ± s.e.m. Standard errors are illustrated as shadowed areas surrounding the plotted averages. The datasets of newly quiescent angles and all angles were statistically distinct (p<10^−12^).

A sleep-like state of the nematode *Caenorhabditis elegans*, which occurs at the end of each larval developmental stage, has been recently described [11]. During this state, termed lethargus, animals reduce their body-curvature and adopt an overall flat posture: the mean body-curvature decreases during the first half of lethargus, increases during the second half of lethargus, peaks at the time of ecdysis when the animal completes the molt by shedding its cuticle, and subsequently returns to its baseline value [12]. In addition, brief alternating bouts of quiescence (stillness – see [11]–[14]) and motion can be readily observed during this period. Interestingly, at least one pronounced body bend often persists even during bouts of complete behavioral quiescence [12], [15], [16]. As a result, the nematodes exhibit a conspicuous hockey-stick-like posture, which is distinctive of lethargus (Fig. 1A). However, the functional benefits of this posture are not well understood.

During lethargus, animals repeatedly rotate rapidly 180° about the longitudinal axis (“flip”), as shown in Fig. 1a and Movies S1-2. Flipping was noted to occur exclusively during lethargus under standard conditions, and was hypothesized to assist ecdysis by mechanically breaking crosslinks between the fibrillar collagen assemblies of the old and the new cuticles in order to loosen the former. Moreover, flipping was observed to preferentially initiate in anterior body-sections, allowing the animal to generate torque at an anterior body bend [17]. If so, the hockey-stick-like posture may functionally assist ecdysis by enabling flips, but neither the behavioral nor the biomechanical aspects of this hypothesis were previously studied systematically. In particular, the conditions under which body-wall muscles that are aligned parallel to the longitudinal axis of the animal could generate torque and drive rotation about the same axis were not previously specified.

Here we show that the hockey-stick-like posture was acquired through characteristic dynamics and was actively maintained. We found that flips occurred preferentially when the animals exhibited a posture with a single body bend, and that when flips were observed, the bend was preferentially in the anterior- or mid-section of the body. In addition, we demonstrate that, in the presence of a body bend, rotation can be the consequence of appropriately actuating the body-wall muscles and quasi-statically minimizing the elastic energy of the body.

## Results

### Stereotypical dynamics characterized the entrance to a quiescent posture

Our previous analysis of the lethargus-specific posture [12], [15] revealed that during bouts of behavioral quiescence body-curvature was reduced, and animals typically exhibited 1–2 body bends (see also Fig. 1A). However, the dynamics of body-posture at the transition into a quiescence bout were not previously characterized. We measured the local angles between 18 pairs of intervals along the midline of the body, and determined whether each of the intervals was individually quiescent at each time-point (see Materials and Methods).

Do different regions of the body become quiescent in a particular order during the transition from motion to quiescence? To address this question we focused on periods of transition from motion to quiescence and measured the magnitude of newly quiescent angles: we grouped all instances when the number of quiescent angles increased by one, i.e., when an animal with n quiescent angles switched to having (n+1) quiescent angles by virtue of an additional body-angle seizing its motion. The mean magnitude of these newly quiescent angles was compared to the mean magnitude of all 18 angles along the body of the animal at the same time-point. The resulting newly quiescent angles and average angles are presented in Fig. 1B as a function of the number of quiescent body-angles (n). Surprisingly, we found that the newly quiescent angles were initially larger than the average body-angle, indicating that animals preferentially initiated the transition into a quiescence bout by freezing positions along the body where the curvature was high (see Movie S3). Gradually, lower-curvature intervals became quiescent and some of the initially frozen body bends could slowly relax to a flat configuration. Typically these stereotypical dynamics ended with 1–2 body bends that persisted throughout the quiescence bout. We concluded that the postures during quiescence bouts were achieved through a temporally coordinated motor program.

### Body bends are actively maintained during lethargus quiescence bouts

**Figure 2:**
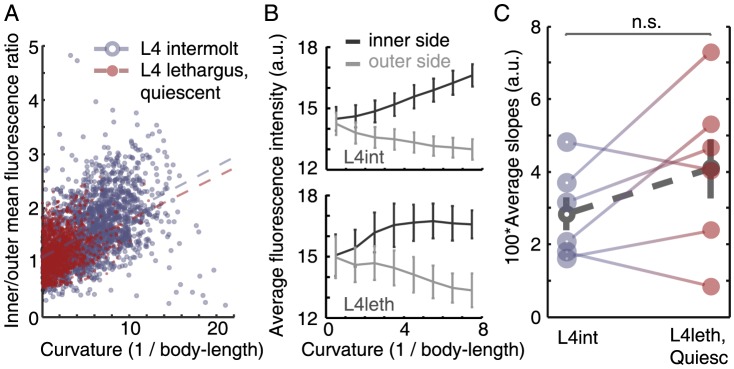
Bends are actively maintained during lethargus quiescence bouts. (A) Values of curvature at all positions along the body were plotted against the ratio of fluorescence of a calcium reporter expressed in the inner and outer body-wall muscles at the same bend. All points plotted were measured from a single animal during L4int (blue, empty circles) and during L4leth bouts of quiescence (red, filled circles). Dashed lines denote the linear correlations between the curvature and the ratio of fluorescence during L4int (blue) and L4leth (red). In this example, the slope of the linear fit during L4int was similar to that during L4leth, indicating a similar relation between body curvature and GCaMP fluorescence in the two developmental states. (B) The absolute values of GCaMP fluorescence that were measured at the inner and outer sides of the body bend during L4int (top) and L4leth (bottom). Similar mean values were obtained at the two stages (N = 6 animals, error bars depict ± s.e.m). (C) Pairwise comparisons of the slopes obtained from N = 6 animals, all analyzed as demonstrated in panel (A). The slopes during L4int and L4leth were not significantly different (p>0.2), indicating that static body bends during quiescence bouts were actively maintained. Dashed line (grey) depicts mean ± s.e.m.

By definition, during quiescence bouts, which can persist for up to several minutes, all body bends are static [12], [15]. Static body bends could be maintained by active muscle contraction (responding to internal cues and interactions with the environment), or passively, e.g. due fully to mechanical interactions with the environment or in analogy to the “catch state” in mollusks, where intracellular free calcium concentration remains at resting level [18]. To distinguish between these possibilities, we used a genetically encoded calcium reporter expressed in the body-wall muscles of wild-type animals and measured the fluorescence at the site of individual body bends in fourth stage larvae (L4). Each animal was sampled both during the intermolt period (L4int) and during lethargus (L4leth), and the fluorescence ratio between the inner- and the outer- muscle groups (as defined by the direction of the bend) were plotted against the curvature at the bend. A typical dataset from an individual animal was plotted in Fig. 2A. Moreover, similar absolute values of fluorescence as a function of body curvature were observed during L4int and L4leth, validating the ratio of intensities as an indicator of muscle activity (Fig. 2B). As expected, the curvature of the body bends could reach higher values during L4int than at the L4leth stage. However, the average increase in the ratio of fluorescence for a given increase in curvature was not significantly different between the L4leth and L4int stage (p>0.2), as seen in Fig. 2C. Taken together with the well established relation between high calcium levels and muscle contraction in motile animals, the similar relation between the calcium levels and degree of muscle contraction indicated that, similar to body bends during L4int, static body bends during quiescence bouts were actively maintained.

### Flips are primarily initiated from a single-bend posture

**Figure 3:**
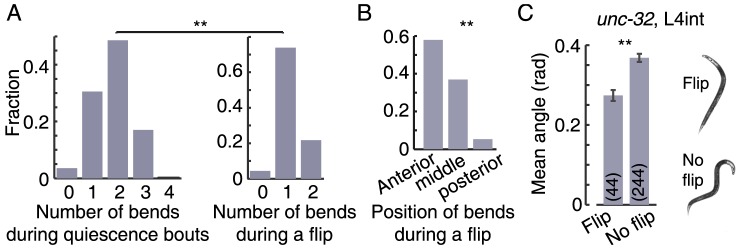
Flips preferentially occur during quiescence bouts with one bend. (A) Left: during quiescence bouts, animals typically exhibit 1–3 body bends. Right: the number of observed body bends during the initiation of successful flips. If the flips were performed independently of the overall body-posture then we would expect the distribution of bends at flip-initiation to mirror the one measured during quiescence bouts in general. In contrast, we found that the two distributions were significantly different (asterisks denote p<0.01), and that the majority of flips were initiated from a single bend posture. (B) When a flip was generated, the position of the bend along the body was preferentially in the anterior- and mid-body regions. The distribution of positions was found to be significantly different from a uniform distribution (asterisks denote p<0.01). Data for all histograms was taken from N = 22 animals during the first half of L4leth. (C) Left: the mean absolute body angles of late L4int *unc-32* mutants, calculated for 100 sec intervals (see methods). Error bars depict ± s.e.m and the numbers of 100 sec intervals that were averaged in each case is denoted in parenthses (asterisks denote p<0.01). Right: two examples of postures, one from an interval in which a flip was detected (top) and the other from an interval in which a flip was not detected (bottom).

During quiescence bouts, animals typically exhibited 1–3 body bends (Fig. 3A). Flips were only observed when the animals were quiescent and only during a small fraction of the quiescence bouts. In order to determine whether flips were preferentially initiated from a subset of the postures of quiescent animals, we measured the number of observed flipping events as a function of the number of body bends immediately prior to each flip. If the flips were independent of the body-posture then we would expect the distribution of postures measured at the time of flip-initiation to mirror the one measured generally during quiescence bouts. In contrast, we found that the two distributions were significantly different (p<0.001); at the times when flips were initiated, the posture of the animal typically exhibited a single body bend (Fig. 3a). In these cases, the bend was equally likely to be ventral or dorsal. We note that the range of observed single bend postures includes localized bends in an otherwise straight body, as well as more gradual bends. Interestingly, the distribution of positions of body bends during flips was significantly different from a uniform distribution (p<0.001). When a flip was generated, the position of the bend along the body was preferentially in the anterior- or mid-body regions, and only in 1 out of 22 cases was it in the posterior region of the body (Fig. 3b). Moreover, when a flip was initiated in the presence of two body bends, the posterior of the two bends often straightened during the flip, consistent with the flip being driven by the anterior bend.

Could an appropriate posture enable flips outside of lethargus? The *unc-32* gene, encoding an ortholog of a subunit of vacuolar proton-translocating ATPase (V-ATPase), was shown to express in the nervous system [19], [20]. Although these mutants are considered coilers based on their adult phenotype, during the late L4int stage they frequently adopt low curvature postures with 0–2 body-bends. We noticed that at this time *unc-32* larvae (occasionally) exhibit flipping behavior. We thus divided the last 2 hours of L4int into 100 sec intervals and compared the mean absolute body-angles between intervals in which we detected a flip and intervals in which we did not (see methods). As shown in Fig. 3C, we found that during intervals that contained flips the posture of the animals was less curved, but not completely straight. Taken together, these findings suggested that the single body bends were functionally required for performing flips, and therefore the characteristic lethargus posture plausibly assists ecdysis.

### A body bend can facilitate a rotation about the longitudinal axis of the animal that is driven solely by contractions of muscles parallel to the axis of rotation

In the absence of body bends, the rotation of a straight nematode about its longitudinal axis would involve generation of azimuthal forces by the body-wall muscles, which is implausible in light of the longitudinal orientation of these muscles [21]. In contrast, if the body of the animal is bent and confined to lie in the plane, differential actuation of its longitudinal muscles coupled to the body-curvature can result in rotation. Rather than preventing rotation altogether, the confinement to the plane, e.g., by a liquid film on an agarose gel or by the boundaries of a microfluidic chip, would map out-of-plane rotation to a flip. The hockey-stick-like posture, distinctive of lethargus, may have a mechanical purpose in facilitating flips.

**Figure 4:**
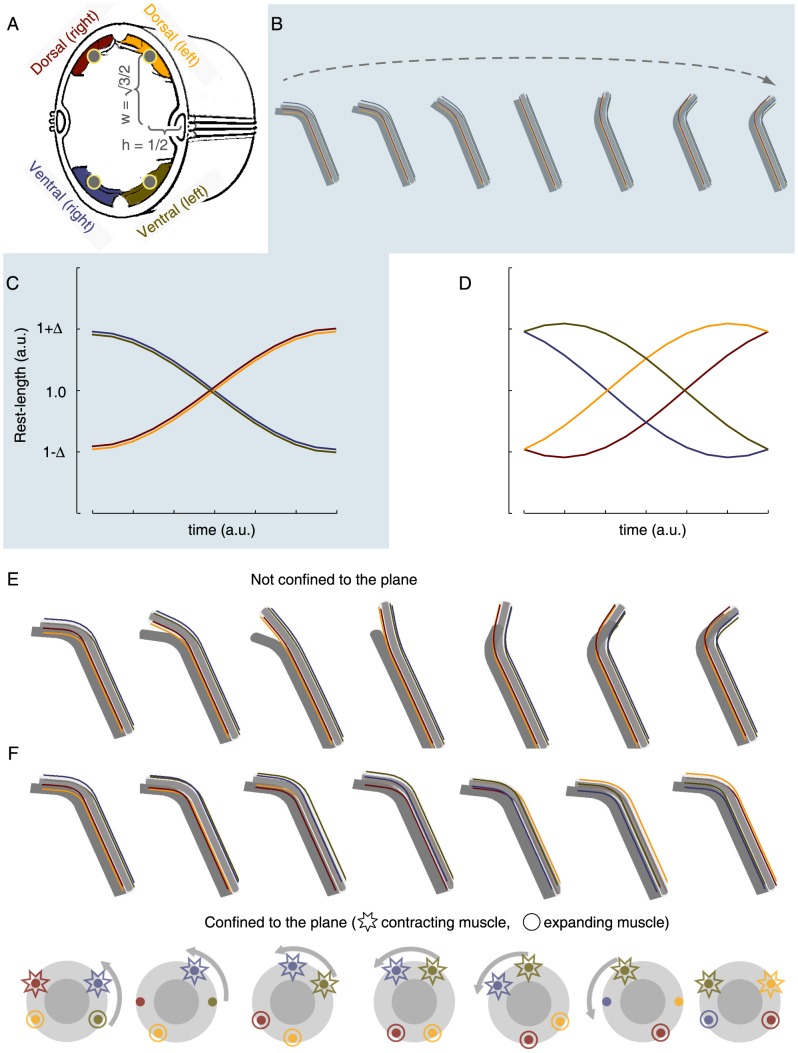
Elastic rod and springs model of *C. elegans* flipping. (A) A schematic drawing of a cross-section of *C. elegans* (adapted from wormatlas.org). The blue/green (ventral) and red/yellow (dorsal) areas denote the cross sections of the four muscle groups. The grey circles located at the center of the muscles denote the positions of the springs that were used in the model (where each row of springs corresponded to a subquadrant row of muscles). (B) Snapshots of the equilibrium postures that corresponded to a dorsoventral swing. Note that the two subdorsal (subventral) quadrants are always in phase with each other (see also Movie S4). (C) The rest-lengths of the four subquadrant springs, located at the region of the body bend, as a function of time during a dorsoventral swing. Dorsoventral swings were achieved by actuating the two ventral subquadrant springs synchronously and the two dorsal subquadrant springs synchronously, but preserving an opposing phase between ventral and dorsal springs. Maximal strains between Δ = 5% and 20% corresponded to common experimentally observed postures. (D) The rest-lengths of the four subquadrant springs, located at the region of the body bend, during a rotation. Rotations were achieved by actuating all four subquadrant springs out of phase. The relative phases of contraction and expansion were chosen such that the magnitude of the curvature remained constant, but the direction of the curvature rotated with respect to the positions of the springs. For instance, the blue/green colored springs were initially located at the outer side of the bend, and arrived finally at the inner side. (E) Snapshots of the equilibrium postures that corresponded to actuating the springs as described in (D), without confining the rod to the plane. This resulted in an out-of-plane rotation (see also Movie S5). (F) Top: Snapshots of the equilibrium postures that corresponded to actuating the springs as described in (D), while confining the rod to the plane. This resulted in a flip (see also Movie S6). Bottom: the equilibrium orientations of a cross-section during a flip. Consecutive snapshot are rotated by π/6 rad relative to each other. Stars and circles indicate contracting and relaxing muscles, respectively. Muscles that are neither contracting nor relaxing, corresponding to extrema in the plot in panel (D), are not emphasized by either shape.

To test this idea, we modeled the nematode as an elastic rod. The framework of viscoelastic rod models has been used extensively to account for the biomechanical properties of the body of *C. elegans* in models of its undulatory locomotion patterns [22]–[28]. We asked whether the minimal model of an elastic rod, when combined with the observation that during a flip the ventral (and dorsal) pairs of muscles at the bend acquire different lengths, would suffice for generating the observed rotation. To answer this question, springs representing the body-wall muscles were placed in the anatomical positions and orientations corresponding to the four muscle groups (Fig. 4). The dimensions and curvature of the rod were given values in the experimentally measured ranges. We then mimicked muscle contraction by differentially modifying the rest-lengths of the springs at the region of the bend, constraining them to follow the profile of rest-lengths that were inferred from observed flips. After each differential step we allowed the system to assume the configuration that would minimize its total energy (see Materials and Methods for details). To most closely resemble our experimental observations, we implemented an actuation profile that resulted in a constant curvature-magnitude but varying curvature-direction. However, this constraint can be relaxed.

In-phase actuation of the dorsal pair of muscle rows and of the ventral pair of muscle rows at the region of the body bend resulted in dorsoventral bending (Fig. 4B–C and Movie S4). When the right sub-ventral muscle row lagged behind the left sub-ventral muscle row and the left sub-dorsal muscle row lagged behind the right sub-dorsal muscle row, the resulting motion was an out of the plane rotation about the longitudinal axis of the rod (Fig. 4D–E and Movie S5). When the same pattern of actuation was combined with planar confinement, i.e. when the body was forced to remain in the plane by an external constraint, the out-of-plane rotation mapped to an in-plane rotation of the rod about its longitudinal axis while the overall spatial posture was maintained, i.e., the rod performed a flip (Fig. 4D, F, and Movie S6). Taken together, our findings demonstrated that a simple biomechanical mechanism is sufficient for driving flips, conditioned on the existence of a body bend.

## Discussion

We have shown experimentally that the posture associated with lethargus-quiescence was acquired using stereotypical dynamics. If quiescence bouts were the mere consequence of the increased stiffness of the double cuticle during molting (an argument that is also inconsistent with the reversibility of locomotion upon stimulation) then the curvature in strongly bent body-intervals would be expected, on average, to relax before or concurrently with less curved body intervals. In contrast, when quiescence bouts were being established, body bends froze first, maintaining their high curvature, and slowly relaxed only later in the process. Once a quiescence bout has been entered, the posture of the animal often included one or two body bends. At the regions of these quiescent bends, the linear relation between the calcium concentration in the body-wall muscles and the degree to which they were contracted was indistinguishable from that of traveling bends during intermolt periods. This indicated that static bends were actively maintained, as opposed to muscle-tension being maintained passively in analogy to the “catch state” in mollusks [18], [29], [30].

The hockey-stick-like posture, although ubiquitous during lethargus and distinctive to this period, was not the one most commonly observed (Fig. 3A). Nevertheless, flips were primarily initiated from this family of single-bend postures, and we constructed a simple biomechanical model to explain these observations. For a straight nematode, all directions are equivalent. This symmetry is broken when a body bend is introduced, singling out the direction of curvature. By appropriately controlling its intrinsic curvature the animal can utilize this broken symmetry to create the torque that would drive its swivel around its longitudinal axis.

Not all biological phenomena have a function in and of themselves; some may be merely byproducts of history or other processes. Flips were proposed to assist ecdysis by loosening the old cuticle [17] for two reasons: (i) they are more abrupt than the typical maneuver performed by *C. elegans*; (ii) they produce lateral strains on the crosslinks between the old and the new cuticle, in contrast to longitudinal locomotion. We show that the hockey-stick-like posture is acquired through a stereotypical motor program and that it allows longitudinally aligned body-wall muscles to generate flips. A functional role for this posture offers a simple, unified, explanation for all of these observations.

Locomotion patterns characteristic of intermolt and adult periods include forward locomotion, backward locomotion, acute (“omega”) turns, and dwelling [15], [31]–[33]. All of these involve generation and propagation of dorsoventral body bends, which is achieved by activating the left and right subventral (and subdorsal) muscle quadrants in-phase, i.e., by adjacent quadrants being matched in their lengths and their activity (contraction or relaxation). In other words, intermolt and adult locomotion patterns could, in principle, be achieved with two, rather than four, arrays of body-wall muscles, one ventral and the other dorsal. Such in-phase activation was noted to be consistent with the observation that the axons of ventral (dorsal) motoneurons synapse onto muscle arms from both the left and the right subventral (subdorsal) quadrants [32], [34]. In contrast, flips necessarily require breaking of the symmetry of ventral and dorsal muscle actuation. Importantly, the bends that enabled flips were preferentially positioned in the anterior of the body and mediated by the contraction of body-wall muscles. Bends were not observed in the head and neck regions, which remained straight during flips (see Fig. 1). Thus, during flips the subventral (and subdorsal) muscle quadrants contract in an out-of-phase fashion; either their lengths or their activity are mismatched (Fig. 4d).

Quasi-equilibrium models, i.e. models that equilibrate after every (small) step, cannot provide information on dynamics. Doing so would require a more detailed, realistic model. For instance, inhomogeneities in the mechanical properties of the animal body are likely to contribute to the dynamics of the flip, but not to the geometrical requirements for flipping. Similarly, the values of mechanical properties may affect the dynamics of flips, but are largely inconsequential to the broad geometric considerations. The relative contributions of these properties to the total energy may affect the results in some cases and we chose parameter values that approximately maintained these relative contributions viz-à-viz published measurements [22]–[28]. In addition, we verified that fine-tuning of these values was not required for flipping. Since the experimental data presented here was insufficient for resolving the dynamics of individual flips, our mechanically homogeneous model captures the appropriate level of complexity.

The observed difference between the lengths if the two subventral (and subdorsal) muscle quadrants is most apparent at the middle of the flip, when the body has rotated by π/2 radians relative to its initial position. When this configuration is observed, the left and right quadrants are positioned on opposing side of the midline such that one is on the outer side of the body-bend and the other is on the inner side. Thus, despite the innervation of pairs of quadrants by single motoneurons, during lethargus each quadrant must be controlled independently. Although compartmentalized activity has been observed in *C. elegans* interneurons [35], indicating its attainability, the phenomenon has not been observed in motoneurons. Additional possible mechanisms for the functional asymmetry of left and right quadrant activation include differential propagation of depolarization from motoneurons through left/right muscle arms or differential intrinsic excitability of left vs. right muscles. The resolution of our experimental data was insufficient for distinguishing between these possibilities.

The preferential initiation of flips when bends were positioned in the anterior- or mid-body indicated that the quadrants in the posterior region of the body were less easily decoupled during lethargus. We previously found that, during L4leth quiescence bouts, body bends were approximately 1.5- and 2-fold more likely to be observed in the mid-body as compared to the anterior and the posterior parts of the body, respectively [12]. This (weak) non-uniformity in the position of bends cannot, in itself, account for the distribution reported in Fig. 3c. To the best of our knowledge, indications of differences in the function of body-wall muscles based on their positions along the body were not previously reported.

Singh and Sulston reported that, although flips were not normally observed during intermolt periods, they could sometimes occur after abusive handling [17]. Taken together with our findings, this observation raises the intriguing possibility that the coupling of adjacent quadrants of body-wall muscles is not merely an unavoidable consequence of the anatomical arrangement of neuromuscular junctions. Rather, there may be a tunable mechanism, plausibly internal to the motoneurons, that couples adjacent quadrants in both the active wakefulness and the quiet wakefulness states during intermolt and adult periods [15]. Such coupling may malfunction as a result of cellular damage or stress. Importantly, it can be downregulated during lethargus, the sleep-like state of *C. elegans*, thus enhancing the repertoire of locomotion patterns of the animal.

## Materials and Methods

### Strains

*C. elegans* strains were maintained and grown according to standard protocols [36]. The following strains were used: *C. elegans* variety Bristol, strain N2 (wild-type) and ZW495 N2; Ex[*Pmyo-3::GCaMP3*].

### Behavioral assays

When appropriate, animals were synchronized by restricting the duration of egg-laying, and assayed in PDMS “artificial dirt” chambers as previously described [12], [15]. Distributions of flips were determined manually by watching a 5× sped up movie of the first hour of L4 lethargus. For every bout, we recorded the number of bends and whether or not the worm flipped. During the first half of lethargus, flips occurred in approximately 2.5% of bouts (during the second half, flips were more frequent but the bouts become less sharply defined). In order to determine whether specific parts of the body were individually quiescent the midline of the animal was found using custom scripts [15] and divided into 20 non-overlapping (tiling) intervals of equal lengths. Similar to the analysis described in [15], 18 positions along the body were associated with the 18 angles, each defined by a pair of next-nearest-neighboring intervals. The temporal dynamics of each of these angles was used to determine whether the corresponding position along the body was quiescent. In brief, using a 5 megapixels CCD camera (Prosilica GC2450, Allied Vision Technologies, Stadtroda, Germany) and a 4.2× magnification, quiescence of an individual angle was defined by the rate of change of the angle not exceeding a threshold of 0.01 radians/sec. Motion detected in an isolated single frame was not considered to interrupt quiescence.

### Scoring flip-containing intervals in *unc-32* mutants outside of lethargus

The last 2 hours of L4int were divided into 100 sec intervals. By visually inspecting the posture of the animal at the first and last frame of each interval we determined whether an odd number of flips occurred during that time. Because the overall rate of flips was low, we reasoned that 100 sec intervals containing no flips or a single flip would contribute most significantly to this dataset. The absolute values of all 18 body-angles were averaged to yield the mean absolute body angle for each frame, which corresponded to the overall body-curvature at a single time-point. Mean absolute angles were then averaged separately for intervals that were flagged as containing flips and intervals that were not.

### Calcium imaging

*Pmyo-3::GCaMP2* late L4 larvae were transferred to PDMS chambers as above, and recorded at a magnification of 20× using a Nikon Eclipse Ti microscope and an Andor iXon ×3 EMCCD camera, both before and after the onset of L4 lethargus. Fluorescence intensity along two sides of worms was determined using a custom Matlab script. First, the boundaries of the body of the animal were located using the Sobel edge detection method [37]. Subsequently, the mean curvature of the inner and the outer boundaries (relative to the direction of the bend) were determined. A mask constructed by a dilation of each edge interval was applied to the original fluorescent image, and the mean intensity of the pixels in each interval was used to calculate the inner/outer fluorescence intensity ratio.

### Statistical analysis

A Mann-Whitney U test was sufficient to demonstrate that the two datasets plotted in Fig. 1B were statistically distinct. A Wilcoxon signed rank test was used to compare the pairwise differences in Fig. 2B. A Mann-Whitney U test was used to compare the two distributions in Fig. 3A and to compare the distribution in Fig. 3B to a uniform distribution. A two-tailed student t-text was used to determine whether the two means in Fig 3C were significantly different.

### Numerical simulation

We investigated numerically the mechanical aspects of *C. elegans* flipping by modeling the animal as a set of linear chains of springs, representing the muscles, connected to an incompressible thin elastic rod, representing the passive body core. Points in the body were described using a curve, ***r*** *(s)*, corresponding to the midline of the rod, and cross-sections perpendicular to ***r*** *(s)* at regular intervals along the rod. Each cross-section defined a planar frame of reference and was assumed to be rigid, such that cross-sections could change their relative positions but not individually deform. Thus, given its position along the midline, each cross-section was fully specified by a unit vector, ***Nˆ***, prescribing its spatial orientation (Fig. 4A).

Two distinct terms could contribute to changes in ***Nˆ***: (1) a change in the direction of the unit tangent vector, ***tˆ*** *= ∂_s_* ***r***, given by the curvature of the mid-line, *κ = *|*∂_s_* ***tˆ***| [38], and (2) a rotation of the cross-section about the tangent vector, quantified by a rate of rotation, *τ = *|*∂_s_* ***Nˆ*** *×* ***tˆ***|. The elastic energy of an isotropic rod with a circular cross-section as a function of these contributions is given by



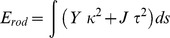
where *Y* is the bending stiffness of the rod and *J* is its twisting stiffness [39].

Each of the four muscle rows was modeled as a chain of twenty-five linear springs and each spring could be separately actuated. The unit vectors ***Nˆ*** (parallel to the dorsoventral axis) and ***Bˆ*** *=* ***tˆ*** *×* ***Nˆ*** (parallel to the left-right axis) spanned the plane of the cross-section. We placed four vertices in each cross-section at (***r*** *±w*, ***Nˆ*** *±h* ***Bˆ***) corresponding to the anatomical left and right subventral and subdorsal quadrants (Fig. 4A). Vertices in the same quadrant from adjacent cross-sections were connected by linear springs of spring constant, *K*, and a variable rest length, *ι^0^*, such that their energy, summed on all springs, was given by 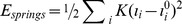



This energy term gave rise to a bending stiffness which was anisotropic, with a maximum at *Y_springs-max_ ∼ w^2^ ι^0^ K*. The intrinsic bending stiffness of the rod was taken to be small compared to the bending stiffness arising from the spring chains *Y ≪ Y_springs-max_.* As a result, the body-posture was fully dictated by the muscle rest-lengths (note that this assumption can be relaxed without qualitatively changing the results). In addition, a high value was used in the model for the torsion stiffness, corresponding to the rigidity of the cuticle of the animals and the incompressibility of its pressurized interior.

The springs were actuated by changing their rest lengths. We fixed the rest-lengths of the anterior 3 quadruplets of springs and posterior 17 quadruplets of springs. The five remaining springs in each of the four chains were given the same rest lengths at any time point. After differentially varying the rest-lengths, the elastic structure modeling the body was relaxed to its new equilibrium position. This was achieved by numerically minimizing the total energy, *E_total_  =  E_rod_+E_springs_+E_confinement_*, using a standard Matlab minimizer (Mathworks Inc., Natick, MA). When appropriate, confinement to a plane was imposed via a harmonic potential 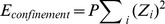
where *Z_ i_* was the Z-component of the center of mass of each of the cross-sections. The numerical values of the parameters used were:



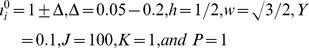



## Supporting Information

Movie S1 **An L4leth** ***C. elegans*** **larvae performing a flip.** The movie was slowed down 20X.
(AVI)

Movie S2 **An animal expressing the fluorescence calcium indicator GCaMP in its body-wall muscles during a bout of quiescence that was interrupted by a flip, shown at 10× real-time.**
(AVI)

Movie S3 **The body midline (black line) and the position of the head (red circle) of an L4leth** ***C. elegans*** **larvae entering a bout of quiescence.** Regions of the animal body that exhibit quiescence are labeled in cyan. A bout of complete behavioral quiescence is established when all body regions are quiescent.
(AVI)

Movie S4 **A dorsoventral swing of the thin elastic rod that resulted from actuating the springs at the subdorsal (subventral) quadrants in phase, as detailed in** **Fig. 4** **.**
(AVI)

Movie S5 **An out of plane rotation of the rod resulting in actuating the different quadrant springs asynchronously, as detailed in** **Fig. 4** **.**
(AVI)

Movie S6 **A flip resulting from confining the rod to the plane and actuating the springs in the same manner as in Movie S5.**
(AVI)
